# COVID-19’s impact on primary care and related mitigation strategies: A scoping review

**DOI:** 10.1080/13814788.2021.1946681

**Published:** 2021-07-20

**Authors:** Jayleigh Lim, John Broughan, Des Crowley, Brendan O’Kelly, Ronan Fawsitt, Mary Carmel Burke, Geoff McCombe, John S. Lambert, Walter Cullen

**Affiliations:** a School of Medicine, University College Dublin, Dublin, Ireland; b Health Services, Addiction Services, Dublin, Ireland; c Mater Misericordiae University Hospital, Dublin, Ireland; d Castle Gardens Surgery, Kilkenny, Ireland; e Ireland East Hospital Group, Dublin, Ireland; f Glasnevin Family Practice, Dublin, Ireland

**Keywords:** Infectious diseases, general practice/family medicine, general, public health and community medicine, health care organisation and management

## Abstract

**Background:**

The COVID-19 pandemic has had a substantial impact on primary care throughout Europe and globally.

**Objectives:**

This review aims to ascertain how the pandemic has impacted primary care service provision/patients and to examine strategies to mitigate these impacts.

**Methods:**

The scoping review framework comprised a six-stage process developed by Arksey and O'Malley. The search process was guided by the Joanna Briggs Institute three-step search strategy and involved searching the PubMed, Embase, Scopus, CINAHL Plus, and Cochrane Library databases. The review is reported according to the Preferred Reporting Items for Systematic Reviews and Meta-Analyses extension for Scoping Reviews. A thematic analysis approach by Braun and Clarke was used to interpret the findings.

**Results:**

Thirty-two studies from 18 countries and six continents were included, 13 reported original research, three were reviews, and 16 were case reports reporting healthcare systems’ experiences of dealing with the pandemic. Emerging themes concerned the COVID-19 pandemic’s impact on primary care service provision and patients, the impact of the rapid transition to telemedicine due to COVID-19 on primary care, and strategies to mitigate the impact of COVID-19 on primary care (i.e. infection prevention and control measures, alternatives/modifications to traditional service delivery or workflow, government policy responses, and education).

**Conclusion:**

The COVID-19 pandemic has considerably impacted on primary care at both service and patient levels, and various strategies to mitigate these impacts have been described. Future research examining the pandemic’s ongoing impacts on primary care, as well as strategies to mitigate these impacts, is a priority.

KEY MESSAGES

- COVID-19 has substantially impacted primary care throughout Europe and globally, and various strategies have been implemented to address this crisis.
- Going forward, primary care must adopt a united, resilient, and adaptive pandemic response. This response must be aided by requisite standards of evidence, financial, and regulatory supports.

## Introduction

The COVID-19 pandemic has had a profound impact on global health and placed unprecedented burden on healthcare systems [1–3], with drastic measures taken by many countries to curtail spread of the disease [4]. To increase the emergency and intensive care capacity, many routine healthcare resources have been diverted to address COVID-19, with non-COVID-19 primary care and speciality care services for chronic and non-urgent care in hospitals largely downscaled or suspended [5].

Due to asymptomatic spread and COVID-19’s high transmission rate, most countries have implemented strict measures to curtail spread of the disease, making regular patient-physician visits challenging. Coupled with patient reluctance towards attending in-person visits due to fears of contracting COVID-19 [5,6], this has contributed to a potential reduction in primary care and outpatient consultations for non-COVID-related chronic conditions such as cancer and diabetes [7,8]. Evidence from prior epidemics suggests that neglect of usual care can be an unintended consequence of prioritising the emergency response, resulting in increased morbidity and mortality related to other causes [9,10]. While there is increasing literature on patients’ short and long-term outcomes with COVID-19 infection [11–15], there is comparatively little evidence documenting the pandemic’s impact on primary care and its patients. This lack of evidence alarming as primary care is where most patients with COVID-19 infection and/or concerns are likely to be treated, and where the pandemic’s long-term consequences will be managed.

Therefore, it is imperative that primary care responds to challenges associated with the COVID-19 pandemic in a constructive way. Thus, this study will seek to inform future practice and research by examining the COVID-19 pandemic’s impacts on primary care at both service and patient levels, as well as strategies employed to mitigate these impacts.

## Methods

A scoping review of the current literature was performed to examine the impacts of COVID-19 on primary care service provision and patients, as well as strategies to mitigate these impacts. Scoping review methods were selected because the manner in which the COVID-19 pandemic has impacted primary care, and the nature of its response, is unclear, and scoping review methods are well suited to investigating topics such as this requiring exploratory yet rigorous mapping of key concepts, evidence, and research gaps [16]. The adopted scoping review framework was developed by Arksey and O’Malley (2005) [16], with recommendations by Levac et al. [17]. Arksey and O’Malley’s framework was chosen because it is a standardised model that facilitates sufficient methodological flexibility, rigour, and transparency during the scoping review process. The framework involves six stages whereby a research question(s) is formulated, relevant studies are identified, selected, charted, collated, summarised, reported, and experts are consulted (Box 1 for more details of our methodological approach). The study’s methodological approach was also informed by Braun and Clarke’s (2008) [18] ‘Thematic Analysis’ framework. This framework also involves six phases, these being – (1) familiarising yourself with your data (2) generating initial codes (3) searching for themes, (4) reviewing themes; (5) defining and naming themes, and (6): producing the report.

## Results

### Search results

Initial searches yielded 319 studies. Removal of duplicates and screening of titles/abstracts, as well as full texts thereafter resulted in the inclusion of 29 studies deemed relevant to the study’s aims. These, along with three additional studies identified from the reference lists of included studies, gave a final selection of 32 studies for inclusion in this review (please see Figure 2).

###  

#### Description of included studies

##### .

###### Nature of extant literature

All studies identified impacts of the COVID-19 pandemic on primary care service provision/patients, and/or strategies to mitigate these impacts. Of these, 13 reported original research, three were reviews, and 16 were case reports. The studies spanned 18 countries in six continents: Europe, Africa, Asia, Australia, and North and South America. All studies were in English and were published in 2020. Further article characteristics are summarised in Supplementary Table 1.

**Figure 1.**
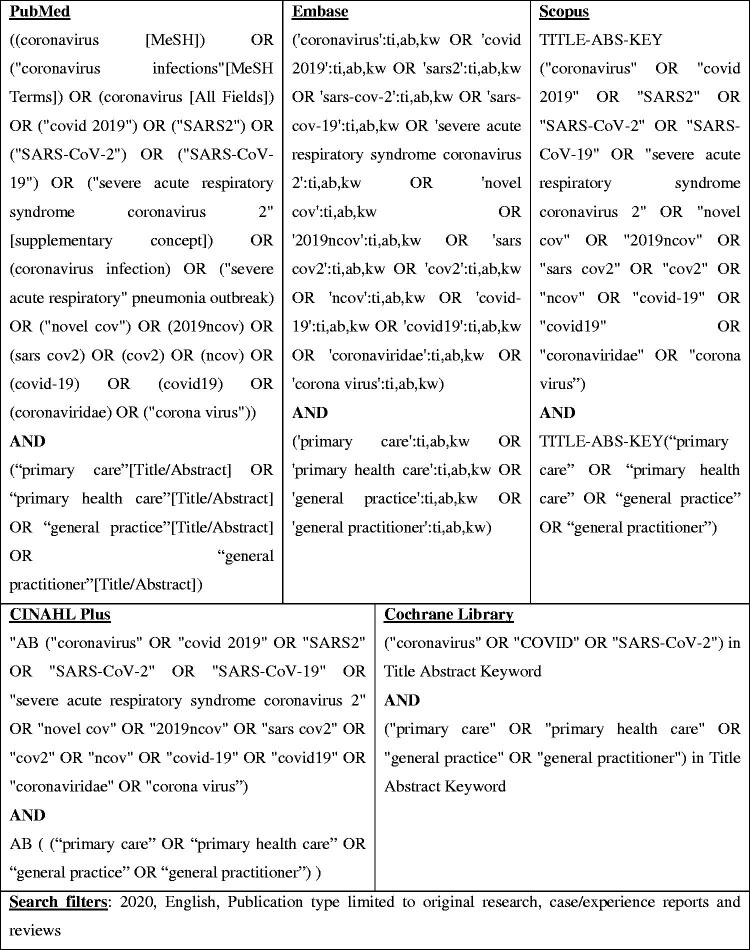
Search strategy.

**Figure 2.**
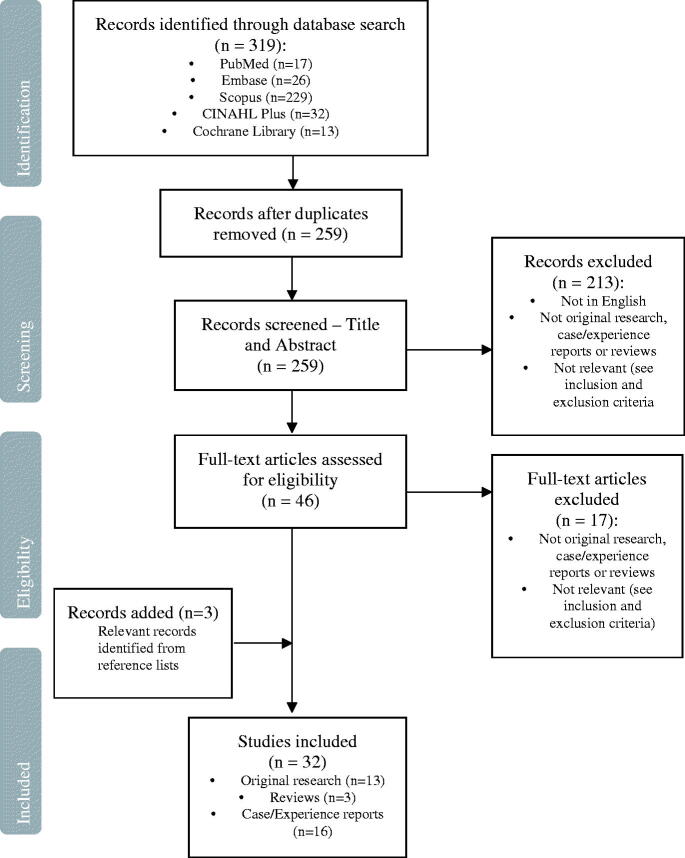
PRISMA-SCR flow diagram.

### Integrated findings

The findings were divided into three main themes. As expected, these themes included (i) the impact of the COVID-19 pandemic on primary care service provision, and (ii) the impact of the COVID-19 pandemic on health outcomes of primary care patients. In addition, another main theme emerged, this being (iii) the impact of the rapid transition to telemedicine due to the COVID-19 pandemic. The studies also identified various strategies to mitigate identified impacts. These strategies were illustrated by one theme: strategies to mitigate the impact of COVID-19 on the community, healthcare provision, and/or patient outcomes. Thematic details are summarised in Supplementary Table 2.

####  

##### Impact of COVID-19 on primary care service provision

Fourteen studies identified the impacts of COVID-19 on primary care service provision [19,22,29–33,35,36,38,42,44,45]. Impacts were classified under the sub-themes: reduced capacity of/access to primary care, reduced quality of primary care, and patients’ avoidance or delaying of non-COVID care.

####  

##### Reduced capacity of/access to primary care

Ten studies documented reduced capacity of/or access to primary care during the pandemic [22,29–33,35,36,38,42]. Reduced capacity was owing to overwhelming numbers of COVID-19 patients and staff shortages [29,31,33], restrictions on in-person consultation opportunities [22,33], and personal protective equipment (PPE) shortages [31,32]. Reduced capacity was also due to transport and logistical difficulties preventing patients from accessing treatments such as medications, dressings, orthoses, and walking aids [29–33], as well as care disruptions for non-COVID-19 services including compromised and/or postponed services for acute care, tuberculosis, diabetic, and HIV patients [30–33,35,36,38,42].

##### Reduced quality of primary care

Four studies identified reduced quality of primary care due to COVID-19 [19,24,30,44]. COVID-19 transmission reduction measures adversely affected clinician-patient relationships [30]. Patient privacy regarding in-person and virtual pharmacy staff/patient interactions was also negatively affected. Privacy was compromised because; (a) pharmacy staff/patients needed to discuss patients’ personal matters more loudly in public spaces due to physical distancing/plastic screens requirements, and (b) insufficient data protection procedures and/or guidelines were in place regarding tele-pharmacy interactions with patients [24]. The primary care continuum of vulnerable patients was also negatively impacted because many patients were confining themselves to home [19]. Additionally, focus on COVID-19 resulted in clinicians struggling to care for chronic conditions, collaborate with medical specialists for non-urgent care, and accurately diagnose non-COVID-19 conditions [30]. Furthermore, some primary care centres were overwhelmed by calls and messages for appointment requests from patients with COVID-19 concerns. These requests caused patient experience to suffer from delayed clinician responding and limited appointment availability [44].

#### Patients’ avoidance or delaying of non-COVID care.

Eight studies identified patients avoiding or delaying non-COVID care due to COVID-19 [22,24,29–31,35,38,45]. Falling consultation rates [22,31,38] during the pandemic were largely due to patients’ COVID-19 infection anxieties [29,30,38] and patients not wanting to ‘waste’ doctors time with non-COVID-19 concerns [30,45].

####  

##### Impact of COVID-19 on health outcomes of primary care patients

Eight studies documented the pandemic’s impact on primary care patients’ health outcomes [19,30,32,33,36,37,39,45]. Impacts were classified into two sub-themes: poorer outcomes in patients with existing comorbidities and poorer mental health outcomes.

##### Adverse outcomes in patients with existing comorbidities

Six studies found adverse health outcomes in patients with existing comorbidities due to the pandemic [19,32,33,36,37,39]. Outcomes were owing to patients’ verified increased risk of contracting COVID-19 and/or experiencing severe illness due to contracting COVID-19 [19,32,36,37,39], as well as reduced access to health services and medications among particularly vulnerable groups (i.e. older, chronic disease, and opioid using populations) [19,22,29,31,36,44]. It was also found that the pandemic may increase burden, as well as health and social inequities in HIV and Tuberculosis populations [22,31].

##### Adverse mental health outcomes

Four studies identified poorer mental health outcomes due to COVID-19 [19,30,36,45]. This trend was largely due to stress caused by the pandemic as well as the consequences of lockdown measures (e.g. breakdown in people’s social networks (especially those of older individuals), intrafamilial violence, substance misuse) [18,36,38]. Pandemic related adverse mental health outcomes included loneliness, anxiety, and depression [18,36,38].

##### Impact of the rapid transition to telehealth due to COVID-19

Six studies documented the rapid adaptation of and impact of telemedicine on healthcare delivery. Studies particularly emphasised its use in conducting remote risk assessment and triaging patients for referral for COVID-19 testing or face to face consultation [21,28,30,34,36,48]. Telemedicine was found to have positive and negative consequences for primary care service delivery.

##### Enhanced access to/quality of care

Five studies found the transition to telemedicine enhanced access to/quality of care [21,28,30,34,48]. Telemedicine was favoured for its ability to ensure patient and clinician safety, care continuity (especially for patients who are busy and/or living remotely), as well as good chronic disease management, mental health follow-up, wellness cheques, and medication procedures [18,24,39,45]. The move to telemedicine was also deemed broadly acceptable by patients [24,39,45].

##### Reduced access to/quality of care

Conversely, five studies identified how rapid transition to telemedicine reduced access to/quality of care [21,28,30,34,36]. The move away from in-person visits to telemedicine was found to reduce healthcare opportunities for certain groups, including older patients, people with limited technological access/ability, severe mental illness, and substance abusers [24,31,37]. Meanwhile, telemedicine was found to impair quality of patient care because of logistical issues, patients being unable to receive timely physical examination and other procedures without in-person consultation, privacy, safety, and confidentiality concerns, clinicians experiencing difficulties understanding patients’ needs and with clinical decision-making, and unmet needs for telemedicine related program funding and health insurance coverage [18,24,37,45].

##### Strategies to mitigate the impact of COVID-19 on the community, healthcare provision and/or patient outcomes

Twenty-eight studies identified strategies to mitigate COVID-19’s impact on communities, healthcare provision, and/or patient outcomes [19–28,30–43,45,48–50]. These strategies were classified into the following subthemes: infection prevention and control measures, alternatives/modifications to traditional service delivery or workflow, government policy responses, and education.

##### Infection prevention and control measures

Nine studies identified various infection prevention and control measures to mitigate COVID-19 spread in communities [20,24,30,31,35,45–47,49]. These included triaging patients [20,30,46,47,49], implementing respiratory symptoms clinics to separate COVID/non-COVID work flows [20,30,35], infection prevention and control measures in consultation rooms and waiting areas [30,31,45,49], and providing clinicians with infection prevention and control training [35,46,47].

##### Alternatives/modifications to traditional service delivery or workflow

Twenty-six studies identified alternatives/modifications to convential service delivery or workflow, which mitigated COVID-19 spread in communities, and reduced COVID-19’s impact on healthcare provision and/or patient outcomes [19–25,7,28,30,32–43,45,48–50].

These alternatives/modifications included shifting from in-person to telemedicine consultations. This shift was implemented to ensure patients continue receiving care in the community, reduce practice footfall and reduce pressure on facility-based healthcare systems [19–22,28,30,33–36,38,39,42,43,45,48–50]. Home care was also provided to patients deemed unsuitable for telemedicine or requiring in-person care [32,38,45,49], and outreach services were implemented to screen and care for vulnerable patients [19,30,32,40,45]. Mobile health applications were also used in some health systems to help clinicians provide care during the pandemic [25,27,48].

Measures were also adopted in community pharmacies to minimise unnecessary exposure to COVID-19 while ensuring patients receive their medications. These included transitioning from paper to electronic prescriptions [24,35,39], increased use of self-service dispensing lockers or special medication pick-up counters [24], medication home deliveries [24,32,35,37], multi-month dispensing [32], and working in fixed shifts of pharmacy technicians [24].

In some health systems, tools were developed and implemented in response to unmet needs arising during the pandemic. For instance, the ‘Evaluation SOcio-GERiatrique’ (ESOGER) [42] and 2019-nCoV 3I [48] tools have been used to help clinicians identify people in the community at risk of COVID-19 infection and/or adverse health consequences of COVID-19 related home confinement [19]. Meanwhile, the innovative population management approach [20] and integrating an electronic health record note template within primary care workflows [41] have also contributed to more timely and efficient preventative and treatment-based management of COVID-19 patients in primary care.

Other primary care modifications include enhancing collaboration between primary care and medical specialists (i.e. psychologists and psychiatrists) and providing family-focussed behavioural interventions [30,36], as well as hospice and palliative services in response to lockdown related health problems [49].

##### Government policy responses

Four studies reported on government policy responses developed to mitigate COVID-19’s impact on healthcare provision and, subsequently patient care [30,31,35,48]. These included emergency plans to optimise primary care’s COVID-19 response and telemedicine consultations *via* regulatory and financial supports [18,30,39]. Other government measures were also proposed, including efforts to secure adequate personal protective equipment (PPE) and rapid diagnostic tests. Efforts were also made to implement improved public health policy and enhance coordination between public and private primary healthcare [51].

###### Education

Five studies identified education as a strategy to mitigate COVID-19’s impact on healthcare provision and/or patient outcomes [26,30,31,33,49]. These included patient education initiatives concerning health advice on COVID-19 and infection control practices, as well as home-based self-care of chronic conditions [30,31,49]. Clinician education was also provided to meet clinical demands of the pandemic [29].

## Discussion

### Main findings

The findings show that the pandemic has impacted primary care globally, and that its effects on service provision and patient health have mainly been adverse. Various efforts have been made to mitigate COVID-19’s impact on primary care. These efforts primarly focussed on providing remote care, controlling COVID-19 spread in communities, managing patients affected by COVID-19 infection, government supports for primary care and COVID-19 related educational initiatives for primary care patients and professionals. However, bar the exception of telemedicine initiatives which were shown to have mixed outcomes for primary care service provision and patients, the extent to which adopted mitigation strategies have succeeded is unclear, and so future evaluations of these initiatives are warranted.

### How the findings relate to other literature

This study’s findings largely support those of existing literature on the topic. However, we believe this study has added value because it provides a comprehensive and detailed account of how the COVID-19 pandemic has impacted primary care services and patients worldwide. The study also has added value because it allows for a detailed description, and at times evaluation, of strategies implemented to mitigate these impacts. Previous studies have shown that the pandemic has led to substantial and widespread reorganisation of primary care [1–5], and this study’s findings overwhelmingly support these views. Notably, this study found that most services worldwide seek to provide care as safely and remotely as possible using various infection prevention and control strategies, some more successfully than others. These measures include social/physical distancing, rapid testing and diagnostics, educational interventions, PPE, and telemedicine. However, previous research has also shown that these strategies, whilst necessary, have had adverse effects on primary care patients throughout the world [5–8], and this study’s findings also support these views. In particular, this study demonstrated that the reorganisation of primary care has often led to reduced access to and quality of care for patients, thus resulting in adverse health consequences (e.g. mental health issues, reduced access to medications) for many patients, and perhaps most gravely for vulnerable patients including those with existing health conditions, older populations, domestic violence victims, and people with severe psychological and/or addiction issues.

### Implications for clinical practice

This review’s findings suggest that while healthcare needs, policy, structures, and resources vary in different countries, primary care strategies adopted in response to the COVID-19 pandemic are largely similar. Hence, there is potential for health systems to share recommendations, best practices, lessons learnt, and strategies to adapt to and thrive in the challenging and evolving healthcare landscape amid a pandemic. Further, this study’s findings suggest that going forward, primary care must exercise greater flexibility, resilience, and responsiveness to optimise patient outcomes and to enhance service provision during COVID-19 and future pandemics. While various mitigation strategies have already been implemented worldwide, there is a need to evaluate these strategies to inform future practice and increase primary care preparedness during similar crises. The rapid transition to or expansion of telemedicine has been a particularly important mitigation strategy. While reports of its effectiveness during the COVID-19 pandemic have been mixed, studies suggest that its use is likely to persist after the pandemic. If so, primary care telemedicine resources must be supported by requisite standards of evidence, funding, and data protection legislation.

### Future research

Scoping reviews aim to identify gaps in the literature [52]. Doing so in this review has been challenging because there is much that we do not know about the pandemic’s impact on primary care and its patients. Thus, identifying the most prominent and important knowledge gaps has been difficult. For example, we found that while considerable research has focussed on COVID-19 patients’ short and long-term clinical outcomes [11–15], there remain much fewer studies examining the pandemic’s impact on primary care and its patients. Therefore, future research should make ongoing efforts to monitor impacts of this nature. Such research should also focus on evaluating strategies to mitigate the pandemic’s effect on primary care. Remote care initiatives, transmission prevention and control measures, outreach services, assessment tools, and governmental and educational interventions have all been applied to mitigate the COVID-19 pandemic’s effects. Future research should further investigate the effectiveness of all these strategies. This study’s findings also suggest that of all these strategies, our knowledge of telemedicine approaches is the most complete, and reports of its usefulness in primary care have been mixed. However, this study also found that the frequent application of telemedicine is likely to continue in primary care, even after the COVID-19 pandemic has passed. So future research evaluating these methods further may be particularly merited.

### Methodological considerations

This study benefitted from adopting several established methodological guidelines, frameworks, databases, tools, and techniques, including Arksey and O’Malley’s (2005) scoping review framework [16], Levac et al.’s (2010) revisions to this framework [17], the Joanna Briggs Institute three-step search strategy (2015) [51], the PubMed, Embase, Scopus, CINAHL Plus, and Cochrane Library databases, the PRISMA-SCR, and Braun and Clarke’s (2008) thematic analysis approach [18]. However, this study also had some limitations. For instance, while we strived to be comprehensive in our approach, there is a possibility that our search strategy identified not all publications relevant to the inclusion criteria, choice of electronic databases and hand searching process (e.g. grey literature). Conversely, we acknowledge that greater efficiency in the literature searching process may have been achieved had we used narrower search terms. Narrower terms may have yielded fewer search results, resulting in us needing to exclude fewer studies than we did. In addition, as is tradition with scoping reviews, no assessment of study quality was undertaken as part of this review. We focussed on covering the range of work that informs the topic rather than limiting ourselves to studies that meet the highest standards of scientific rigour. Further, only studies published in English were considered for inclusion in our review. This approach may have resulted in the exclusion of equally relevant literature published in other languages.

## Conclusion

This review provides an in-depth description of how the COVID-19 pandemic has impacted primary care service provision and primary care patients. Moreover, this review has added value because it provides a comprehensive and detailed account of strategies implemented to mitigate these impacts. Going forward, primary care services must adopt a united, adaptive, and evidence-based approach to managing challenges presented by the COVID-19 pandemic.

## Supplementary Material

Supplementary Table 2

Supplementary Table 1
