# Supplementary material for: COVID-19’s impact on primary care and related mitigation strategies: A scoping review
Source: Eur J Gen Pract. 2021 Jul 20;27(1):166–75. doi: 10.1080/13814788.2021.1946681 (PMC8293960; doi:10.1080/13814788.2021.1946681)
Supplement: Supplementary Table 2 [file IGEN_A_1946681_SM7249.docx]

**Supplementary Table 2. Summary of themes explored*.***

| **Theme** | **Subthemes** | **Papers exploring theme/subtheme** |
| --- | --- | --- |
| **Impact of COVID-19 on primary care service provision** | **Reduced capacity of/ access to primary care** | - **Joy et al., 2020 [21]**: Fall in face-to-face consultation rates, home visits, and clinical administrative appointments - **Silva-Tinoco et al., 2020 [22]**: Restrictions to stay home made it difficult to access pharmacological treatment; patients stopped attending their routine medical appointments in health centres due to the lack of capacity of overwhelmed healthcare system by COVID-19 patients - **Verhoeven et al., 2020 [23]**: Chronic care is mostly postponed; Prevention not linked to COVID-19 is not a priority for most GPs; Screening activities are suspended; Some diagnostics such as non-urgent radiology are not available - **Yu et al., 2020 [24]**: Primary care services adjusted non-acute hospital services and/or reduced consultation times; Lack of PPE and/or rapid tests in primary care resulting in inability to provide clinical services - **Jain et al., 2020 [25]**: Considerable disruption in all aspects of TB service provision (prevention, surveillance, treatment); Non-COVID-19 health services such as TB surveillance hampered; Suspension of TB immunization services; PPE shortage make it impossible to provide safe regular healthcare for TB patients - **Jaly et al., 2020 [26]**: Provision of face-to-face care for patients with DFD made difficult by the need to protect vulnerable and shielded groups; Decreased capacity; Staff shortages and staff-related sickness; Reduced allied health professional input; Transport difficulties Logistical difficulties - **Andrikopoulos et al., 2020 [27]**: Major disruption and changes to business processes of many allied health services; Reduced access to primary care, diagnostic and hospital services for diabetes led to significant drop in access to usual diabetes care - **Armbruster et al., 2020 [28]**: Disruption to HIV primary care services - **Chamboredon et al., 2020 [29]**: Non-urgent medical activities deprogrammed; Monitoring of chronic pathologies reorganized - **Dimer et al., 2020 [30]**: Suspension of face-to-face speech-language and hearing therapy |
|  | **Reduced quality of primary care** | - **Beauchet et al., 2020 [32]**: Physical distancing may break down the primary care continuum of the older community-dwelling population - **Koster et al., 2020 [34]:** Concerns about the quality of pharmaceutical care, especially for vulnerable patients; Decrease in privacy in the pharmacy due to placement of plastic screens and not being able to use a separate consultation room due to social distance regulations; Pharmaceutical care more distant and the pharmacy less approachable; Patients asked fewer questions; Fewer pharmacotherapy consultation group meetings that served to improve prescribing quality - **Verhoeven et al., 2020 [23]**: Chronic care is dealt with less effectively; Communication is affected in physical consultations because of protective measures taken; Increased risk of missing other diagnoses due to large focus on COVID-19, Less satisfactory collaboration with medical specialists for non-urgent care - **Judson et al.**, **2020 [31]**: Difficulty providing care to patients who needed it most with front-line clinicians busy with triaging the huge volume of calls, messages and appointment requests from patients concerned about COVID-19; Infection control hazard created by surge of patients walking into primary care for advice on COVID-19; Difficulty maintaining consistency in medical recommendation and advice due to rapidly changing information and guidelines; Patient experience suffered with unusually long telephone hold times, delayed message responses, and limited appointment availability; |
|  | **Patients’ avoidance or delaying of non-COVID care** | - **Joy et al., 2020 [21]**: Fall in overall rate of consultations - **Koster et al., 2020 [34]**: Many community pharmacists believe patients postponed their doctor’s visit - **Silva-Tinoco et al., 2020 [22]**: Many patients stopped attending their routine medical appointments in health centres due to the risk of contagion - **Verhoeven et al., 2020 [23]**: Patients consult less frequently for non-COVID care as they fear taking time from their GPs or are afraid of getting infected - **Yu et al., 2020 [24]**: Fewer patients seeking primary care services - **Andrikopoulos et al., 2020 [27]**: Fear of exposure to the virus in primary care, diagnostic and hospital services for diabetes - **Chamboredon et al., 2020 [29]**: Large proportion of population have given up on their usual acute or chronic care due to containment measures and fears of contamination; Number of GP consultations decreased by 44% since the beginning of containment - **Krist et al., 2020 [33]**: Decreased number of patients seeking non-infection-related care |
| **Impact of COVID-19 on health outcomes of primary care patients** | **Poorer outcomes in patients with existing comorbidities** | - **Beauchet et al., 2020 [32]**: Physical distancing may break down the primary care continuum of the older community-dwelling population and increase their risk of visiting EDs and being admitted to hospitals - **Jain et al., 2020 [25]**: Hampered non-COVID-19 health services such as TB surveillance resulting in a drop in diagnosis of new cases of active TB which could potentially result in a surge in number of patients with TB once the lockdown is lifted; Worsening of the increase in TB transmission due to the social, economic and biomedical consequences of the pandemic; TB patients may develop multidrug resistance and superinfection by COVID-19 - **Jaly et al., 2020 [26]**: Combination of reduced physical activity, unhealthy diet and emotional stress during the pandemic can culminate in poor glycaemic control, further increasing the risk of developing DFD - **Brey et al., 2020 [35]**: Patients with chronic disease would be put at risk of COVID-19 if they had to travel and gather in groups to receive medications - **Armbruster et al., 2020 [28]**: Exacerbation of existing health and social inequities that impede successful clinical outcomes and increase HIV disparities; Increased risk of COVID-19 exposure and negative impact on treatment engagement, medication adherence and viral suppression, resulting in disease progression and increased HIV transmission - **Crowley and Delargy, 2020 [36]**: Opioid users particularly vulnerable during COVID-19; Disruption in OAT associated with increased risk of overdose or withdrawal symptoms and reduced likelihood to follow public health advice |
|  | **Poorer mental health outcomes** | - **Beauchet et al., 2020 [32]**: Physical distancing implies home confinement for older community dwellers, thus exposing them to a breakdown in their social networks, and predisposes to psychological fallout - **Verhoeven et al., 2020 [23]**: Loneliness, depression and intrafamilial violence seen more frequently due to lockdown measures - **Armbruster et al., 2020 [28]**: Heightened symptoms of existing anxiety and depression - **Krist et al.**, **2020 [32]**: Increased mental health needs and substance misuse |
| **Impact of the rapid transition to telemedicine due to COVID-19** | **Enhanced access to/ quality of care** | - **Holtz, 2020 [37]**: Patients were overall satisfied with their telemedicine experience, with past users being more satisfied than new users; Telemedicine allowed patients to avoid waiting rooms and risk of getting sick - **Srinivasan et al., 2020 [38]**: Virtual visits ensures safety of patients and providers and ensures access to care, enhances access for those living far away or who are busy, unveiled a new way of practicing medicine, and encourages patients to be more accountable and check themselves at home - **Verhoeven [23] et al., 2020**: Several GPs proactively telephone their chronic patients if they are unable to do home visits or see them in their office; Phone consultations are now reimbursed by health insurance - **Wynn [39] et al., 2020**: Shift to e-health during the initial phase of the pandemic appeared to be seen as acceptable or even welcomed - **Olayiwola [40] et al., 2020**: Acceptable by patients with high engagement, High patient satisfaction, Applicable for a wide range of primary care needs |
|  | **Reduced access to/ quality of care** | - **Holtz, 2020[37]**: New telemedicine users **preferred to see their provider in person** more than past users - **Srinivasan et al., 2020[38]**: The rapid switch to virtual visits resulted in some logistical issues and required adapting to; Concerns that critical patient care issues were unattended or missed; Reduced access for some patients, particularly the elderly; Concerns with issues of privacy, safety and confidentiality; Sustainability hinges on continued program funding - **Verhoeven et al., 2020 [23]**: Loss of non-verbal communication, the limited ability of some patients to articulate their needs, intercultural communication and associated language problems result in difficulties in understanding patients’ needs and the fear of missing important diagnoses; Less information can be obtained in telephone consultations, making clinical decision-making more difficult; Telephone consultations are often not sufficient for acute psychological care - **Wynn et al., 2020 [38]**: Lack of traditional services particularly challenging for some patient groups such as severely mentally ill patients or patients with substance use problems; Inability to receive timely physical examination and other procedures that necessitate physical consultations; Patients may not be able to fully benefit from the provider-patient relationship without physical face-to-face encounters - **Armbruster et al., 2020 [27]**: Transition to telemedicine unreliable for many patients with limited access to technology |
| **Strategies to mitigate the impact of COVID-19 on the community, healthcare provision, and/or patient outcomes** | **Infection prevention and control measures** | - **Blazey-Martin et al., 2020 [41]**: Dedicated on-site respiratory symptoms clinic; Nursing phone triage algorithm - **Koster et al., 2020 [34]**: Changes in logistical procedures including stricter hygiene protocol, Only allow medication pickup after receiving a message from the pharmacy - **Verhoeven et al., 2020 [23]**: Telephone triage; ‘Corona centres’ initiated by local GPs’ teams and organised within the structure of existing ‘out-of-hours’ General Practice Cooperatives to separate COVID and non-COVID flows; Reduction in face-to-face consultations; Infection control measures: removal of unnecessary materials in waiting or consultation rooms and limiting number of patients in the waiting room - **Yu et al., 2020 [24]**: Changes in infection control practices in clinics in response to COVID-19 outbreak - **Andrikopoulos et al., 2020 [27]**: General practice-led respiratory clinics; Online infection prevention and control training - **Krist et al., 2020 [33]**: Promote physical distancing, handwashing and limiting contact - **Lim and Wong, 2020 [51]**: Triaging patients who present to clinics; Prompt updating and training of clinical staff on latest infection control measures; Adequate PPE provision for staff protection; Strict enforcement of compliance to PPE guidelines - **Mills et al., 2020 [52]**: Home-based primary care-led outbreak mitigation involving an Outbreak Preparedness and Action Committee, Web application for COVID-19 triage and reporting, Mobile symptom-screening application, educational training and ongoing COVID-19 mitigation guidance - **Oseni et al, 2020 [48]**: Triaging patients who present to clinics, Infection control measures in place in clinic and waiting areas |
|  | **Alternatives/ Modifications to traditional service delivery or workflow** | - **Beauchet [32] et al., 2020** : ESOGER short assessment tool to screen older community dwellers to identify those with COVID-19 and those at risk of home confinement-related adverse consequences; Connect vulnerable older community-dwelling patients with telemedicine to connect them with their family physician and/or the senior autonomy support program - **Blazey-Martin et al., 2020 [41]**: Shift in-person appointments to telemedicine visits; Innovative population management approach to help remotely manage COVID-19 patients recovering at home, indicate when a visit to the respiratory symptoms clinic was required, and provide timely alerts to instances when additional urgent interventions were required - **Holtz, 2020 [37]**: Telemedicine - **Joy et al., 2020 [21]**: Increase in telephone consults and electronic/video consultations, particularly amongst older adults who were frail or receiving polypharmacy; Digital-first approach to accessing primary care to reduce total footfall into practices, protecting both staff and patients. - **Koenig et al., 2020 [42]**: 2019-nCoV 3I Tool to assist primary care clinicians in effectively managing persons with suspected or confirmed 2019-nCOV - **Koster et al., 2020 [34]**: Electronic prescriptions instead of paper prescriptions, Medicine self-service dispensing lockers or special medication pick-up counters, Home delivery of medication, Patient education and counselling via provision of additional written information or referral to online information to reduce duration of patient encounters, Medication reviews by telephone - **Liu et al., 2020 [43]**: DDC19 mobile-based decision support system for COVID-19 to help GPs collect data, dynamically assess risks, and effectively triage, manage and follow-up patients during the outbreak - **Sossai et al, 2020 [44]**: *Davinci Salute* mobile health application providing an all-in-one solution, from health monitoring to treatment-driven data, for patients seeking primary care anywhere and at any time - **Srinivasan et al., 2020 [38]**: Conversion of all urgent and primary care patient visits to virtual visits - **Verhoeven et al., 2020 [23]**: Primary contact with patients by telephone; GPs to proactively anticipate problems in and reach out to vulnerable and frail patients; Enhanced collaboration in primary care with psychologists and psychiatrists and some medical specialists - **Jain et al., 2020 [25]**: Telemedicine via video link or tele-conferencing to reduce pressure on facility-based healthcare systems and help in community management of TB; Home visits by healthcare workers wearing the appropriate PPE to follow-up patients not suited for video nor teleconferencing; Multi-month dispensing of anti-TB medications to ensure provision of treatment and minimise unnecessary exposure to COVID-19; Outreach services to reach patients with TB; Postal delivery of TB medications - **Jaly et al., 2020 [26]**: Telemedicine consultations for remote monitoring of DFD - **Wynn et al., 2020 [39]**: E-health services including video consultations - **Andrikopoulos et al., 2020 [27]**: Telemedicine consultations to ensure continuity of care; Electronic prescriptions; Medication delivery services - **Brey et al., 2020 [35]**: Home delivery of medication - **Armbruster et al., 2020 [28]**: Rapid scale up of telemedicine; Coordinated delivery of case management and mental health services - **Chamboredon et al., 2020 [29]**: Telecare management by nurses in primary care; Face-to-face monitoring at the patient’s home if deemed necessary - **Crowley and Delargy, 2020 [36]**: National model of remote care for accessing and providing OAT treatment that involves initial telephone triage, remote video assessment, and delivery of scripts to a designated local community pharmacy - **David and Mash, 2020 [45]**: Community screening and testing programme which links public health and primary care approaches, carefully maps cases in vulnerable communities, target screening around cases, test those that screened positive, and provide health education and linkage to primary care - **Deeds et al., 2020 [46]**: Integrate an electronic health record note template into primary care workflows to screen for COVID-19, track cases and guide outpatient care - **Dimer et al, 2020 [30]**: Telemedicine delivery of speech-language and hearing therapy via telephone video - **Grange et al**.**, 2020 [47]**: Telemedicine to screen patients before they presented in person or screen them remotely - **Krist et al.**, **2020 [33]**: Increase virtual visits and telephone-based care; Implement proactive population care to remain in touch with patients; Primary care-led “home hospital” care - **Olayiwola et al., 2020 [40]**: Virtual health – remote monitoring, store-and-forward technology, mobile health applications, direct patient telemedicine care - **Oseni et al., 2020 [48]**: Telemedicine, Home-based care with appropriate protective measures taken; Provision of family-focused behavioural interventions, Provision of hospice and palliative services - **Spelman et al.**, **2020 [49]**: Rapid transition to virtual care |
|  | **Government policy responses** | - **Verhoeven et al., 2020 [23]**: The Belgian government rolled out an **emergency plan for general practice** - **Yu et al., 2020 [24]**: Surveyed family physicians suggested government/local health authorities-instituted measures to facilitate their COVID-19 response: Securing adequate PPE supply, More effective public health policy to contain the outbreak, Setting up a Primary Care Authority to enhance coordination between public and private primary healthcare, Introduction of designated clinics and rapid diagnostic tests - **Andrikopoulos et al., 2020 [27]**: The Australian Government provided COVID-19 specific support to primary and specialist care services by establishing a national call centre, supporting general practice-led respiratory clinics and providing online infection prevention and control training; Facilitattelemedicine consultations by broadening access to its universal access scheme, the Medicare Benefits Scheme and removed certain requirements for reimbursement - **Olayiwola et al., 2020 [40]**: Telemedicine regulatory changes |
|  | **Education** | - **Roberts et al., 2020 [50]:** Outpatient Palliative Care Toolkit to educate primary care clinicians on advance care planning and end-of-life symptom management for vulnerable patients in primary care - **Verhoeven et al., 2020 [23]**: Provide and repeat COVID-19 health advice - **Yu et al., 2020 [24]**: Public education on infection control practices and reporting accurate travel and contact history during consultations - **Jaly et al**., **2020 [26]:** Patient education and the use of online resource for DFD care; Encouragement of self-examination of feet and regular foot care - **Oseni [48] et al.**, **2020** : Patient education and counselling on COVID-19 |
