# Supplementary material for: COVID-19’s impact on primary care and related mitigation strategies: A scoping review
Source: Eur J Gen Pract. 2021 Jul 20;27(1):166–75. doi: 10.1080/13814788.2021.1946681 (PMC8293960; doi:10.1080/13814788.2021.1946681)
Supplement: Supplementary Table 1 [file IGEN_A_1946681_SM7210.docx]

Supplementary Table 1. Descriptive characteristics of final included studies

| **Authors, Year, Country** | **Study design** | **Population** | **Findings: COVID-19 Impact on patients attending primary care and/or Strategies to mitigate them** |
| --- | --- | --- | --- |
| Telemedicine for housebound older persons during the Covid-19 pandemic | | | |
| Beauchet et al., 2020 [32]  Canada | Design and development study | Older community dwellers with COVID-19 | Impact:   - **Break down of primary care continuum, increase their risk of visiting EDs and being admitted** to hospitals - Home confinement exposes them to a **breakdown in their social networks**, with ensuing p**sychological fallout**   Strategies:   - **ESOGER** (Evaluation Socio-GERiatrique) short assessment to **screen older community dwellers** to identify those with COVID-19 and those at risk for home confinement-related adverse consequences by assessing five subdomains: COVID-19 symptomatology, Frailty, Psychological stress, Social isolation, and Caregiver burden - **Connect those at risk** with **telemedicine** – family physician and/or senior autonomy support program |
| Primary Care Population Management for COVID-19 Patients | | | |
| Blazey-Martin et al., 2020 [41]  US | Design and development study | SARS-CoV-2 positive patients (n=305) of a hospital-based primary care practice | Strategies:   - **Innovative population management approach** including an algorithm, an EMR component, and a twice daily population report that (1) helps **remotely manage COVID-19 patients** recovering at home, (2) **indicate when a visit to the respiratory symptoms clinic was required** without taxing the ED, and (3) **provide timely alert** to instances when additional urgent interventions were required - Shift in-person appointments to **telemedicine visits** - **Nursing phone triage algorithm** to manage high call volume from patients with questions about the coronavirus and concerns about getting tested - **On-site respiratory symptoms clinic** |
| Patients Perceptions of Telemedicine Visits Before and After the Coronavirus Disease 2019 Pandemic | | | |
| Holtz, 2020 [37]  US | Cross-sectional study (survey) | Patients (n=434) who have used telemedicine | Strategies:   - **Telemedicine**   - (+) Patients were **overall satisfied with their telemedicine experience**, with past users being more satisfied than new users   - (+) New users more strongly agreed that they used telemedicine to **avoid waiting rooms and risk getting sick**   - (-) New users **preferred to see their provider** **in person** more than past users |
| Reorganisation of primary care for older adults during COVID-19: a cross-sectional database study in the UK | | | |
| Joy et al., 2020 [21]  UK | Cross-sectional database study | Patents ages ≥65 registered with GPs | Impact:   - **Fall in face-to-face consultation rates** by 64.6%, in home visits by 62.6%, in clinical administrative appointments by 23.6% - **Telephone consults increased** by 106.0% and electronic/video consultations by 102.8% in the same period - Overall, the **rate of consultations dropped** by 27.1% - **Polypharmacy and Frailty were associated with a relative increase in both face-to-face and telephone consultations**   Strategies:   - **Digital-first approach to accessing primary** **care** to reduce total footfall into practices, protecting both staff and patients |
| 2019-nCoV: The Identify-Isolate-Inform (3I) Tool Applied to a Novel Emerging Coronavirus | | | |
| Koenig et al., 2020 [42]  US | Design and development study | Patients with suspected or confirmed 2019-nCOV | Strategies:   - **2019-nCoV 3I (Identify-Isolate-Inform) Tool** to assist emergency and primary care clinicians and out-of-hospital providers in effectively managing persons with suspected or confirmed 2019-nCOV |
| Impact of the COVID-19 epidemic on the provision of pharmaceutical care in community pharmacies | | | |
| Koster et al., 2020 [34]  The Netherlands | Cross-sectional study (questionnaire) | Community pharmacists (n=208), pharmacy technicians (n=6) and pharmacy manager (n=1) | Impact:   - Many respondents (76.7%) expressed **concerns about the quality of pharmaceutical care** especially for vulnerable patients - Majority of the respondents (93.0%) also believed patients **postponed their doctor’s visit** - **Decrease in privacy** in the pharmacy due to placement of plastic screens and not being able to use a separate consultation room - **Pharmaceutical care was more distant and the pharmacy less approachable** - Respondents (60.6%) also felt **patients asked fewer questions** - **Fewer pharmacotherapy consultation group meetings** between GPs and pharmacists to improve prescribing quality   Strategies:   - Follow **stricter hygiene protocol** - Preference for **electronic prescriptions** over paper prescriptions; Increased use of **medicine self-service dispensing lockers or special medication pick-up counters** and Increased **delivery of medication** to the patient’s home; Only allow **picking up of refill medication after a patient receives a message** from the pharmacy; **Working in fixed shifts of pharmacy technicians** - Changes in **patient education and counselling** with shorter **pharmacy encounters; Patients provided with additional written information (e.g. flyers) or referred to online information** (website, video animations) - Changes in **communication with prescribers**: **Medication reviews and contact with prescribers by telephone** |
| A COVID-19 Risk Assessment Decision Support System for General Practitioners: Design and Development Study | | | |
| Liu et al., 2020 [43]  China | Design and development study | General practitioners | Strategies:   - **DDC19, a mobile-based decision support system for COVID-19** to help GPs collect data, dynamically assess risks, and effectively triage, manage and follow-up patients during the COVID-19 outbreak |
| COVID-19 Pandemic Response: Development of Outpatient Palliative Care Toolkit Based on Narrative Communication | | | |
| Roberts et al., 2020 [50]  US | Design and development study | Primary care clinicians | Impact:   - Urgent need for **support in advance care planning and end-of-life symptom management** for vulnerable patients in primary care   Strategies:   - **Palliative care education for primary care clinicians** focused on advanced care planning communication skills and comfort care symptom management at the end of life |
| Telemedicine and the 2019 coronavirus (SARS-CoV-2) | | | |
| Sossai et al., 2020 [44]  Italy | Preliminary report | Possible SARS-CoV-2 patients | Strategies:   - ***Davinci Salute*, a mobile health application** that allows people to video call or chat with a doctor about their symptoms via their smartphone. The app provides an all-in-one solution, from thorough health monitoring to treatment-driven data, for patients seeking primary care anywhere, at any time, thus supporting the healthcare system in the management of possible patients |
| Qualitative Assessment of Rapid System Transformation to Primary Care Video Visits at an Academic Medical Center | | | |
| Srinivasan et al., 2020 [38]  US | Qualitative study (interviews) | Key stakeholders (n=53) of a primary care video visit program | Strategies:   - Conversion of all urgent and primary care patient visits to **virtual visits** – video visits and other remote technologies.   - (+) Ensures safety of patients and providers and ensures access to care   - (+) Enhanced access for patients living far away or who are busy   - (+) Unveiled a new way of practicing medicine   - (+) Encourages patients to be more accountable and check themselves at home   - (-) Logistical issues associated with rapid transition required adapting to   - (-) Concerns that critical patient care issues were unattended or missed   - (-) Reduced access for some patients, particularly the elderly;   - (-) Concerns with issues of privacy, safety and confidentiality   - (+) Sustainability hinges on continued program funding |
| Effect in self-care behaviour and difficulties in coping with diabetes during the COVID-19 pandemic | | | |
| Silva-Tinoco et al., 2020 [22]  Mexico | Cross-sectional study (survey) | Patients (n=212) in primary care with T2DM | Impact**:**   - Restrictions to stay at home made it **difficult to access pharmacological treatment** - Many patients **stopped attending their routine medical appointments** in health centres during the lockdown because of either the risk of contagion or the lack of capacity of the overwhelmed healthcare system by COVID-19 patients |
| Impact of the COVID-19 pandemic on the core functions of primary care: will the cure be worse than the disease? A qualitative interview study in Flemish GPs | | | |
| Verhoeven et al., 2020 [23]  Belgium | Qualitative study (interviews) | GPs (n=132) in Flanders | Impact:   - Patients **consult less frequently for regular non-COVID care** (both acute and chronic), with regular consultations decreasing by 70-80% - **Communication is affected in physical consultations because of protective measures taken** - **Chronic problems are dealt with less effectively** as priority is given to COVID-19, patients present less often for follow-up, and consultations and home visits were reduced to a minimum. This are expected to lead to health problems due to suboptimal follow-up. - **Unavailability of some diagnostics; Prevention not linked to COVID-19 not a priority; Screening activities suspended** - **The large focus on COVID-19 increases the risk of missing other diagnoses** - **Loneliness, depression and intrafamilial violence are seen more frequently due to lockdown measures** - **Less satisfactory collaboration with medical specialists for non-urgent care**   Strategies:   - Belgian government rolled out an **emergency plan for general practice**: **Telephone COVID-19**; **Physical triage centres** accessible after telephone triage; ‘**Corona centres**’ initiated by local GPs’ teams and organised within the structure of existing out-of-hours General Practice Cooperatives to separate COVID and non-COVID flows - **Primary contact with patients by telephone; Reduction in face-to-face consultations**   - (-) Loss of non-verbal communication, the limited ability of some patients to articulate their needs, intercultural communication and associated language problems result in **difficulties in understanding patients’ needs** and the **fear of missing important diagnoses**   - (-) Less information can be obtained in telephone consultations, making **clinical decision-making more difficult**   - (-) Telephone consultations often insufficient for **Acute psychological care**   - (+) Several GPs **proactively telephone their chronic patients** if they are unable to do home visits or see them in their office   - (+) **Health insurance reimbursement for phone consultations** - **Infection control measures**: removing unnecessary materials in waiting/consultation rooms, limiting number of patients in waiting room - **Enhanced collaboration**: More intense collaboration in primary care with psychologists, psychiatrists and some medical specialists - **Providing and repeating COVID-19 health advice** - **Proactively anticipate certain problems in vulnerable and frail patient**s in order to help them and coordinate actions where necessary |
| How are family doctors serving the Hong Kong community during the COVID-19 outbreak? A survey of HKCFP members | | | |
| Yu et al., 2020 [24]  Hong Kong | Cross-sectional study (survey) | Family physicians providing primary care services during COVID-19 (n=491) | Impact:   - **Fewer patients seeking primary care services** - Primary care services **adjusted non-acute hospital services and/or reduced consultation time** - **Lack of PPE and/or rapid tests** in primary care resulting in inability to provide clinical services   Strategies**:**   - **Changes in infection control practices** in response to COVID-19 outbreak - Suggested **government/ local health authorities-instituted measures** to facilitate frontline family doctors to respond to the outbreak: Securing adequate PPE supply; More effective public health policy to contain the outbreak; Primary Care Authority to enhance coordination between public and private primary healthcare; Introduction of designated clinics and rapid diagnostic tests; **Public education on** infection control practice and reporting accurate travel and contact history during consultations |
| Tuberculosis in the era of COVID-19 in India | | | |
| Jain et al., 2020 [25]  India | Literature review | Patients with tuberculosis | Impact:   - Considerable **disruption in all aspects of TB service provisions** (prevention, surveillance, treatment): **TB surveillance hampered** resulting in a **drop in diagnosis of new cases** of active TB and a potential surge in number of patients with TB once the lockdown is lifted; **PPE shortage** makes it impossible for healthcare workers to provide safe regular healthcare for patients with TB; **Suspension of TB immunization services** - Social, economic and biomedical consequences expected to worsen the **increase in TB transmission; Potential development of multidrug resistance** and **superinfection by coronavirus**   Strategies:   - **Virtual communication platforms (telemedicine via video link or tele-conferencing)** to reduce pressure on facility-based health-care systems and help in community management of TB - **Home visits** by healthcare workers wearing the appropriate PPE to follow up patients not suited for video nor teleconferencing - **Ensure provision of anti-TB treatment**: **Multi-month dispensing; Outreach services** and **postal delivery of TB medications** |
| Redefining diabetic foot disease management service during COVID-19 pandemic | | | |
| Jaly et al., 2020 [26]  UK, India | Literature review | Patients with diabetic foot disease (DFD) | Impact:   - Difficulties in service provision: **Provision of face-to-face care for patients with DFD made difficult; Decreased capacity; Staff shortages and staff-related sickness; Reduced allied health professional input** - Difficulties faced by patients: **Transport and Logistical difficulties, Patient-related challenges** where a combination of reduced physical activity, unhealthy diet and emotional stress during the pandemic can culminate in poor glycaemic control further increasing the risk of developing DFD   Strategies:   - **Patient education and the use of online resources** for DFD care - **Encouragement of self-examination of feet and regular foot care:** Diabetes monitoring, Offloading and regular foot examination - **Telemedicine consultations** for remote monitoring of DFD |
| E-Health in Norway Before and During the Initial Phase of the Covid-19 Pandemic | | | |
| Wynn, 2020 [39]  Norway | Literature review | Patients using e-health | Strategies:   - **E-health services** including video consultations to reduce the risk of contagion   - (+) Shift to e-health during the initial phase of the pandemic appeared to be seen as **acceptable or even welcomed**   - (-) **Lack of traditional services was challenging particularly for some patient groups**, such as severely mentally ill patients or patients with substance use problems   - (-) Patients **might not receive timely physical examination and other procedures that necessitate physical consultations**, while others **may not be able to fully benefit from the provider-patient relationship without physical face-to-face encounters** |
| The Australian response to the COVID-19 pandemic and diabetes – Lessons learned | | | |
| Andrikopoulos and Johnson, 2020 [27]  Australia | Case/  Experience report | Patients receiving diabetes care | Impact:   - **Major disruption and changes to business processes** of many allied health services - **Reduced access** to primary care, diagnostic and hospital services for diabetes, combined with **fear of exposure to the virus** in these settings, led to a **significant drop in access to usual diabetes care**.   Strategies:   - Australian Government provided COVID-19 specific support to primary and specialist care services by establishing a **national call centre**, **supporting general practice-led respiratory clinics** and providing **online infection prevention and control training**. - Australian Government **broadened access** to its universal access scheme, the Medicare Benefits Scheme and removed certain requirements for reimbursement for **telemedicine** - **Electronic prescription** and **Medication delivery services** |
| Addressing Health Inequities Exacerbated by COVID-19 Among Youth with HIV: Expanding Our Toolkit | | | |
| Armbruster et al., 2020 [28]  US | Case/  Experience report | Youth (aged 13-24) with HIV (n=76) in primary care | Impact   - **Ability to provide intensive, in-person HIV primary care services abruptly disrupted** - **Transition to telemedicine has been unreliable for many patients who have limited access to technology** - COVID-19 could **exacerbate existing health and social inequities** that **impede successful clinical outcomes** and **increase HIV disparities** - YHIV with **existing anxiety and depression** experienced **heighted symptoms** during the pandemic - Increased **risk of COVID-19 exposure** and **negative impact on treatment engagement, medication adherence and viral suppression, resulting in disease progression and increased HIV transmission**   Strategies:   - **Rapid scale up of telemedicine** to counter restrictions to in-person and walk-in visits - **Coordinated delivery of case management and mental health services** |
| Home delivery of medications during Coronavirus disease 2019, Cape Town, South Africa: Short report | | | |
| Brey et al., 2020 [35]  South Africa | Case/  Experience report | Primary care patients with chronic diseases | Impact:   - **Patients with chronic diseases would be put at risk of contracting COVID-19** if they had to travel and gather in groups to receive medication   Strategies:   - **Home delivery of medication** by linking the existing chronic dispensing unit system with the emerging approach to community-orientated primary care in the Metro. Its strengths, weaknesses, opportunities and threats are further discussed. |
| COVID-19 pandemic in France: health emergency experiences from the field | | | |
| Chamboredon et al., 2020 [29]  France | Case/  Experience report | Health systems | Impact:   - **Non-urgent medical activities were deprogrammed**, and the **monitoring of chronic pathologies were reorganized** - Large proportion of the population have **given up on their usual, acute or chronic care** due to containment measures and fear of contamination - **Number of consultations with GPs decreased by 44%** since the beginning of containment   Strategies:   - **Strengthening nursing roles in primary care**: **Telecare** management, fully covered by the French Health Insurance, of patients with COVID-19 by home nurses, with **face-to-face monitoring** at the patient’s home if deemed necessary. |
| A national model of remote care for assessing and providing opioid agonist treatment during the COVID-19 pandemic: a report | | | |
| Crowley and Delargy, 2020 [36]  Ireland | Case/  Experience report | Primary care patients on opioid agonist treatment (OAT) | Impact:   - **Opioid users are particularly vulnerable during this,** especially older drug users - Disruption in OAT associated with **increased risk of overdose and withdrawal symptoms** and **reduced likelihood to follow public health advice**   Strategies:   - **National model of remote care for assessing and providing OAT treatment** involving **Initial telephone COVID-risk triage**, **remote video assessment** and the **safe and timely delivery of scripts to a designated local community pharmacy**. |
| Community-based screening and testing for Coronavirus in Cape Town, South Africa: Short report | | | |
| David and Mash, 2020 [45]  South Africa | Case/  Experience report | Primary health care | Impact:   - Cape town has many **vulnerable communities** due to poverty, overcrowding and comorbidities.   Strategies:   - **Community screening and testing (CST) programme** linking public health and primary care approaches, careful mapping of cases in highly vulnerable communities, targeted screening around cases, testing those that screened positive, health education and linkage to primary care. |
| Leveraging an electronic health record note template to standardize screening and testing for COVID-19 | | | |
| Deeds et al., 2020 [46]  US | Case/  Experience report | Veterans (n-1,338) receiving primary care services | Strategies:   - **Integrate an electronic health record (EHR) note template into primary care workflows** to screen patients for COVID-19 symptoms, track cases, and guide outpatient care |
| The COVID-19 pandemic and the implementation of telemedicine in speech-language and hearing therapy for patients at home: an experience report | | | |
| Dimer et al., 2020 [30]  Brazil | Case/ Experience report | Primary care patients (n=17) receiving speech-language and hearing therapy | Impact:   - **Suspension of face-to-face visits**   Strategies:   - **Telemedicine delivery of speech-language and hearing therapy** via telephone video |
| Responding to COVID-19: The UW Medicine Information Technology Services Experience | | | |
| Grange et al., 2020 [47]  US | Case/  Experience report | Health systems | Strategies:   - **Hospital incident command structure** - **Telemedicine** to either **screen patients before they presente**d in person or **screen them remotely** while isolating the patient in a room with telemedicine capabilities |
| Rapid design and implementation of an integrated patient self-triage and self-scheduling tool for COVID-19 | | | |
| Judson et al., 2020 [31]  US | Case/  Experience report | Primary care patients (n=950) | Impact:   - **Difficulty providing care to patients who needed it most as front-line clinicians were busy triaging** the huge volume of phone calls, patient portal messages and appointment requests from patients with concerns about COVID-19 - **Infection control hazard** creased by the surge of patients walking into primary care practices for advice - **Difficulty maintaining consistency in medical recommendation and advice** due to rapidly changing information and guidelines - **Patient experience suffered** with unusually long telephone hold times, delayed message responses, and limited appointment availability   Strategies:   - **Digital patient-facing self-triage and self-scheduling tool** to provide patients with 24-hour access to personalized recommendations and information regarding COVID-19 and to improve ambulatory surge capacity through self-triage, self-scheduling, and avoidance of unnecessary in-person care |
| Redesigning Primary Care to Address the COVID19 Pandemic in the Midst of the Pandemic | | | |
| Krist et al., 2020 [33]  US | Case/  Experience report | Primary care practices | Impact:   - **Decreased number of patients seeking non-infection–related care**, potentially with adverse consequences - Increased **mental health needs and substance misuse**   Strategies:   - Promote **physical distancing, handwashing and limiting contact** - Increase **virtual visits and telephone-based care**, conversion to nearly complete virtual care, delay non-urgent appointments - Implement **proactive population care** to remain in touch with patients - Primary care-led **“home hospital” care** |
| COVID-19: Notes From the Front Line, Singapore’s Primary Health Care Perspective | | | |
| Lim and Wong, 2020 [51]  Singapore | Case/  Experience report | Private general practitioner clinics (n=50) | Strategies   - **Triaging** patients who present to clinics, **Telephone review of patients**, **Telemedicine mobile application** for remote consultation - **Prompt updating and training of clinical staff** on latest infection control measures**, Adequate PPE provision** for staff protection throughout the pandemic, **Strict enforcement of compliance to PPE guidelines** |
| Home-Based Primary Care Led-Outbreak Mitigation in Assisted Living Facilities in the First 100 Days of Coronavirus Disease 2019 | | | |
| Mills et al., 2020 [52]  US | Case/  Experience report | Residents (n=1,794) of assisted living facilities served by home-based primary care | Strategies:   - **Home-Based Primary Care-led outbreak mitigation** to suppress COVID-19 in assisted living facilitates involving an **Outbreak Preparedness and Action Committee** that developed a comprehensive preparedness plan and served to consolidate internal and external communications regarding COVID-19; Development of a **web application for COVID-19 triage** and reporting; Development of a **mobile-enabled symptom-screening application for employee self-screening** **Infection control measures** adapted from the CDC, **educational training** and **ongoing COVID-19 mitigation guidance** |
| Telemedicine as a Bright Spot of the COVID-19 Pandemic: Recommendations From the Virtual Frontlines (“Frontweb”) | | | |
| Olayiwola et al., 2020 [40]  US | Case/  Experience Report | Primary care clinicians | Strategies:   - **Virtual health**: Remote monitoring, Store-and-forward technology, Mobile health applications, Direct patient telemedicine care (electronic visits, telephone visits, video visits, electronic consults)   - (+) Considerable increase in telemedicine engagement over weeks, with ~93% of care delivered through this platform   - (+) Patient satisfaction remained at pre-pandemic high levels, per very preliminary internal patient experience data   - (+) Applied for a wide range of primary care needs: chronic disease management, well-person care and wellness checks, mental health follow-up, medication management, new patient encounters, acute nonemergent complaints e.g. back pain, headache, and rash, and lifestyle counselling. - **Telemedicine regulatory changes** to address financial and regulatory barriers which hinder telemedicine acceleration and adoption |
| The role of the family physician in the fight against Coronavirus disease 2019 in Nigeria | | | |
| Oseni et al., 2020 [48]  Nigeria | Case/  Experience report | Family physicians | Strategies:   - **Triaging** patients as they present to family medicine clinics by nurses to separate potential COVID-19 patients from other patients - Conducive **clinics and waiting areas** with **infection control measures** in place - **Telemedicine** to minimise need of patients coming to hospital - **Home-based care** with appropriate protective measures taken - Provision of **family-focused behavioural interventions** to prevent health problems arising from the lockdown - **Provision of hospice and palliative care services** to the elderly and terminally ill - **Patient education and counselling** to provide accurate information on how to prevent the disease and what to do if there is a suspected case |
| A Model for Rapid Transition to Virtual Care, VA Connecticut Primary Care Response to COVID-19 | | | |
| Spelman et al., 2020 [49]  US | Case/  Experience report | VA Connecticut Health Care System | Strategies:   - A primary care system model for **rapid transition to virtual care (RTVC)** including **immediate virtual care conversion**, **telework expansion**, **implementation of virtual respiratory urgent care clinics**, and **development of standardized note templates** demonstrates expeditious and sustained transition to virtual care during the COVID-19 pandemic. |
